# Supplementary material for: Phagolysosomes break down the membrane of a non-apoptotic corpse independent of macroautophagy
Source: bioRxiv. 2024 Jun 20:2024.06.19.599770. Preprint. [Version 1] doi: 10.1101/2024.06.19.599770 (PMC11212964; doi:10.1101/2024.06.19.599770)
Supplement: Supplement 1 [file NIHPP2024.06.19.599770v1-supplement-1.pdf]

bioRxiv preprint doi: https://doi.org/10.1101/2024.06.19.599770; this version posted June 20, 2024. The copyright holder for this preprint (which was not certified by peer review) is the author/funder, who has granted bioRxiv a license to display the preprint in perpetuity. It is made available under aCC-BY 4.0 International license.

Table S1. Worm strains.

Strain	Genotype	Source
N2	Wild type	(16)
AZ212	*unc-119(ed3) ruIs32[pAZ132: pie-1p::GFP::H2B; unc-119(+)] III*	(36)
COP93	*ttTi5605 II; unc-119(ed3) III*	InVivo Biosystems
FT97	*cdc-42(gk388) / mIn1[dpy-10(e128) mIs14(myo-2::gfp; pes-10::gfp)] xnIs25[cdc-42::GFP::CDC-42; unc-119(+)] II; unc-119(ed3) III*	Jeremy Nance Lab
FT1056	*lgg-1(tm3489) / mIn1[dpy-10(e128) mIs14(myo-2::gfp; pes-10::gfp)] II*	Crossed JJ1610 to RD
HZ1687	*atg-9(bp564) him-5(e1490) V*	(37)
JJ1610	*pkc-3(ok544)/mIn1[dpy-10(e128) mIs14] II; him-8(e1489) IV*	(38)
OD58	*unc-119(ed3) ltIs38[pie-1p::GFP::PH(PLC1delta1), unc-119(+)] III*	(39)
RB2372	*atg-16.2(ok3224) II*	(40)
RD	*lgg-1(tm3489) / dpy-10(e128) unc-4(e120) II*	(41)
VC20426	*atg-16.2(gk145022[W253*]) II and 519 other mutations*	(22)
VC40503	*atg-16.1(gk668615[Q356*]) X and 693 other mutations*	(22)
VIG25	*Is[pVIG57: Pmex-5::mCherry::LGG-2::tbb-2 3'UTR; C.b. unc-119(+)] II; unc-119(ed3) III*	(2)
WEH80	*mIn1[dpy-10(e128) mIs14(myo-2::gfp; pes-10::gfp)] II; ltIs38[pie-1::GFP::PH(PLC1delta1) unc-119(+)] xnIs8[pJN343: nmy-2::NMY-2-mCherry; unc-119(+)] unc-119(ed3) III; lgg-2(tm5755) IV*	Crossed
WEH82	*lgg-1(tm3489) / mIn1[dpy-10(e128) mIs14(myo-2::gfp; pes-10::gfp)] II; ltIs38[pie-1::GFP::PH(PLC1delta1), unc-119(+)] xnIs8[pJN343: nmy-2::NMY-2::mCherry; unc-119(+)] unc-119(ed3) III; lgg-2(tm5755) IV*	Crossed WEH80 to FT1056
WEH95	*xnIs390[pie-1::GFP::PH(PLCdelta1), unc-119(+)]; unc-119(ed3) III; Is[mCherry::HistoneH2B] IV*	(42)

bioRxiv preprint doi: https://doi.org/10.1101/2024.06.19.599770; this version posted June 20, 2024. The copyright holder for this preprint (which was not certified by peer review) is the author/funder, who has granted bioRxiv a license to display the preprint in perpetuity. It is made available under aCC-BY 4.0 International license.

WEH224	*Si[pVIG57: Pmex-5::mCherry::LGG-2::tbb-2 3'UTR, C.b. unc-119(+)] II; unc-119(ed3) ruIs32[pAZ132: pie-1::GFP::H2B, unc-119(+)] III*	Crossed VIG25 to AZ212
WEH260	*unc-119(ed3) III; wurIs90[pGF7:pie-1p::mCh::PH::ZF1, unc-119(+)]*	(42)
WEH399	*unc-119(ed3) III; wurIs144[pGF13: pie-1::ZF1::mCherry::his-15, unc-119(+)]*	(18)
WEH464	*wurIs90[pGF7:pie-1::mCh::PH::ZF1, unc-119(+)] II; unc-119(ed3) ltIs38[pie-1::GFP::PH(PLC1delta1), unc-119(+)] III*	Crossed WEH260 to OD58
WEH683	*xnIs390[pie-1::GFP::ZF1::PH(PLC1delta1), unc-119(+)] II; Is[mCherry::HistoneH2B] IV; atg-16.1(gk668615[Q356*]) X*	Crossed VC40503 to WEH95
WEH684	*atg-16.2(ok3224) xnIs390[pie-1::GFP::ZF1::PH(PLC1delta1), unc-119(+)] II; Is[mCherry::HistoneH2B] IV*	Crossed RB2372 to WEH95
WEH696	*Strain lost. atg-16.2(ok3224) xnIs390[pie-1::GFP::ZF1::PH(PLC1delta1), unc-119(+)] / mIn1[dpy-10(e128) mIs14(myo-2::gfp; pes-10::gfp)] xnIs25[cdc-42::GFP::CDC-42; unc-119(+)] II; unc-119(ed3) III; Is[mCherry::HistoneH2B] IV; atg-16.1(gk668615[Q356*]) X*	Crossed WEH683 to WEH684, then to FT97
WEH700	*wurIs144[pGF13: pie-1::ZF1::mCherry::his-15; unc-119(+)] I; unc-119(ed3) ltIs38[pie-1::GFP::PH(PLC1delta1), unc-119(+)] III*	Crossed WEH464 to WEH399
WEH707	*wurIs144[pGF13: pie-1::ZF1::mCherry::his-15; unc-119(+)] I; unc-119(ed3) ltIs38[pie-1::GFP::PH(PLC1delta1), unc-119(+)] III; atg-16.1(gk668615[Q356*]) X*	Crossed WEH683 to WEH700
WEH708	*wurIs144[pGF13: pie-1::ZF1::mCherry::his-15; unc-119(+)] I; atg-16.2(gk145022[W253*]) II; unc-119(ed3) ltIs38[pie-1::GFP::PH(PLC1delta1), unc-119(+)] III*	Crossed VC20426 to N2, then to WEH700
WEH709	*wurIs144[pGF13: pie-1::ZF1::mCherry::his-15; unc-119(+)] I; mIn1[dpy-10(e128) mIs14(myo-2::gfp; pes-10::gfp)] II; unc-119(ed3) ltIs38[pie-1::GFP::PH(PLC1delta1), unc-119(+)] III*	Crossed WEH82 to WEH700
WEH711	*wurIs144[pGF13: pie-1::ZF1::mCherry::his-15; unc-119(+)] I; atg-16.2(gk145022[W253*]) II; unc-*	Crossed WEH707 to WEH708

bioRxiv preprint doi: https://doi.org/10.1101/2024.06.19.599770; this version posted June 20, 2024. The copyright holder for this preprint (which was not certified by peer review) is the author/funder, who has granted bioRxiv a license to display the preprint in perpetuity. It is made available under aCC-BY 4.0 International license.

	119(ed3) ltIs38[pie-1::GFP::PH(PLC1delta1), unc-119(+)] III; atg-16.1(gk668615[Q356]) X*	
WEH714	*wurIs144[pGF13: pie-1::ZF1::mCherry::his-15; unc-119(+)] I; atg-16.2(gk145022[W253*]) II; unc-119(ed3) ltIs38[pie-1::GFP::PH(PLC1delta1), unc-119(+)] III; atg-16.1(gk668615[Q356*]) X*	Crossed WEH708 to WEH707 to WEH709
WEH718	*Si[pVIG57: Pmex-5::mCherry::LGG-2::tbb-2 3'UTR, C.b. unc-119(+)] atg-16.2(gk145022[W253*]) II; unc-119(ed3) ltIs38[pie-1::GFP::PH(PLC1delta1), unc-119(+)] III*	Crossed WEH708 to WEH224
WEH722	*Si[pVIG57: Pmex-5::mCherry::LGG-2::tbb-2 3'UTR, C.b. unc-119(+)] II; unc-119(ed3) ruIs32[pAZ132: pie-1::GFP::H2B, unc-119(+)] III*	Crossed WEH224 to N2
WEH728	*Si[pVIG57: Pmex-5::mCherry::LGG-2::tbb-2 3'UTR, C.b. unc-119(+)] atg-16.2(gk145022[W253*]) II; unc-119(ed3) ruIs32[pAZ132: pie-1::GFP::H2B, unc-119(+)] III*	Crossed WEH722 to WEH718
WEH729	*Si[pVIG57: Pmex-5::mCherry::LGG-2::tbb-2 3'UTR, C.b. unc-119(+)] II; unc-119(ed3) ruIs32[pAZ132: pie-1::GFP::H2B, unc-119(+)] III; atg-9(bp564) V*	Crossed HZ1687 to WEH722
WEH731	*wurIs144[pGF13: pie-1::ZF1::mCherry::his-15; unc-119(+)] I; unc-119(ed3) ltIs38[pie-1::GFP::PH(PLC1delta1), unc-119(+)] III; atg-9(bp564) V*	Crossed HZ1687 to WEH700
WEH734	*Si[pVIG57: Pmex-5::mCherry::LGG-2::tbb-2 3'UTR, C.b. unc-119(+)] II; unc-119(ed3) ruIs32[pAZ132: pie-1::GFP::H2B, unc-119(+)] III; atg-16.1(gk668615[Q356*]) X*	Crossed WEH683 to WEH722
WEH739	*Si[pVIG57: Pmex-5::mCherry::LGG-2::tbb-2 3'UTR, C.b. unc-119(+)] atg-16.2(gk145022[W253*]) II; unc-119(ed3) ruIs32[pAZ132: pie-1::GFP::H2B, unc-119(+)] III; atg-16.1(gk668615[Q356*]) X*	Crossed WEH734 to WEH728
WEH751	*wurSi2[pVIG57-CTPD: Pmex-5::CTPD::mCherry::LGG-2::tbb-2 3'UTR, C.b. unc-119(+)] II; unc-119(ed3) III*	Homozygosed from worms injected by InVivo Biosystems
WEH755	*wurSi2[pVIG57-CTPD: Pmex-5::CTPD::mCherry::LGG-2::tbb-2 3'UTR, C.b. unc-119(+)] II; unc-119(ed3) ruIs32[pAZ132: pie-1::GFP::H2B, unc-119(+)] III*	Crossed AZ212 to WEH751

bioRxiv preprint doi: https://doi.org/10.1101/2024.06.19.599770; this version posted June 20, 2024. The copyright holder for this preprint (which was not certified by peer review) is the author/funder, who has granted bioRxiv a license to display the preprint in perpetuity. It is made available under aCC-BY 4.0 International license.

Table S2. Oligonucleotide primers.

**Primer**	**Sequence**	**Genotyped Allele or Purpose**
atg-9 exon 5 F2	CTGGATCACTCGGGTCATCAAA	*atg-9(bp564)*
atg-9 intron 5 R	CCGAATTTCAGGCGATTTTCAGACA	*atg-9(bp564)*
gk668615 HinfI F	GTTATCAAAATTTGGGAgAT	*atg-16.1(gk668615)*
atg-16.1 exon 10R	GATCCGTTGACCTTCCATCGT	*atg-16.1(gk668615)*
atg-16.2 exon 1 F	GGCTGACAGTGAATCTCGTT	*atg-16.2(ok3224),* RT-PCR
ok3224 InnerLeft	CCGTATGGCCAGAAAACGTA	*atg-16.2(ok3224)*
atg-16.2 exon 2 R	CATCTGTGCGCGTTCTTCTT	*atg-16.2(ok3224),* RT-PCR
atg-16.2 exon 2 F3	ACGCAGCTAGATAACGAACGAT	*atg-16.2(gk145022)*
gk145022 HinfI R	GCTGTCGCAAATGTATCATCAGAGA	*atg-16.2(gk145022)*
oJN27	GTCCTTCTTGAGTTTGTAACAGC	*ltIs38*
ZF1 NheI R	CCTGCTAGCCCTCGGAACTCTCAGCTCAT	*ltIs38*
ttTi5605 F2	TGCTTATCTCGAATGAGACCCTT	*Si[pVIG57]*
ttTi5605 R	GACACCCGGGTTTGTCTAGAT	*Si[pVIG57]*
Cb-unc-119N R	GAGCTGGGGAGAAGAAGACACT	*Si[pVIG57]*
tat-5 geno F	TGCTCCAATCACTTACTGGGGAC	RT-PCR
tat-5 geno R	TACGCGGAGTGAAATTGGAATAA	RT-PCR
mex-5p oma-1(219) F	CTCATTGTATTCTCTCTTAATTAATTTTATCGATAATCAATTGAATGTTTCAGACAGAGAATGATTGCCGCTCCCCCACTTTCTG	CTPD subcloning
mCh oma-1(378) R	TTATCTTCTTCACCCTTTGAGACATCAGTTGCGACAGATTTCATCAGAAGAGATTGAGCA	CTPD subcloning
