# Supplementary material for: Phagolysosomes break down the membrane of a non-apoptotic corpse independent of macroautophagy
Source: bioRxiv. 2024 Jun 20:2024.06.19.599770. Preprint. [Version 1] doi: 10.1101/2024.06.19.599770 (PMC11212964; doi:10.1101/2024.06.19.599770)
Supplement: Supplement 2 [file NIHPP2024.06.19.599770v1-supplement-2.pdf]

Fig S1. **ATG-9 and ATG-16 alleles used in this study.**

ATG-9 has four transmembrane domains (TM) and two alpha-helices predicted to be partially embedded in the membrane (M), based on Guardia, Tan (35). Most of the protein is cytosolic, except for two small luminal domains (L). ATG-16.1 and ATG-16.2 have an N-terminal domain predicted to bind ATG-5 and ATG-12, a central coiled-coil domain (CCD) important for macroautophagy, and a C-terminal WD40 domain important for non-canonical autophagy (LAP/CASM). Positions of point mutations and the *ok3224* deletion are indicated.

Fig S2. ***atg-16.2* mRNA levels are reduced by a premature stop codon in the WD40 domain.** (A) Three biological replicates of wild-type N2 and *atg-16.2(gk145022[W253*])* cDNA were co-amplified for *atg-16.2* and *tat-5* as a loading control. (B) Graph of normalized subtracted fluorescence intensity ratios. The *atg-16.2(gk145022[W253*])* mutant band is significantly reduced compared to wild-type using a one-tailed t-test. ***$p<0.001$.

Fig S3. Macroautophagy is not required for polar body internalization.

Timing of polar body internalization after the 4-cell stage. Control embryos averaged 5±2 minutes after the 4-cell stage (n=11). There was no significant delay in internalization in *atg-9* single mutants (5±2, n=10) or *atg-16.2(W253*); atg-16.1(Q356*)* double mutants (6±3, n=10). Data are presented as mean ± std dev. One-tailed t-test, $p>0.3$.

Video S1. LGG-2 accumulates in mitotic nuclei as the nuclear envelope breaks down.

A 2-cell *C. elegans* embryo developing to the 4-cell stage shows the timing of LC3 reporter mCh::LGG-2 accumulation inside the nucleus, as well as to centrosomes and spindle microtubules. Mitotic stage is visible in the merged image with histone GFP::H2B reporter. A z-series was recorded every 45 seconds, six 1.5 µm z-step images were max projected, and the projections are displayed at 5 fps using Imaris.
